# Sensorineural Tinnitus: Its Pathology and Probable Therapies

**DOI:** 10.1155/2016/2830157

**Published:** 2016-02-08

**Authors:** Aage R. Møller

**Affiliations:** The University of Texas at Dallas, School of Behavioral and Brain Sciences, 800 W Campbell Road, Richardson, TX 75080, USA

## Abstract

Tinnitus is not a single disease but a group of different diseases with different pathologies and therefore different treatments. Regarding tinnitus as a single disease is hampering progress in understanding of the pathophysiology of tinnitus and perhaps, more importantly, it is a serious obstacle in development of effective treatments for tinnitus. Subjective tinnitus is a phantom sound that takes many different forms and has similarities with chronic neuropathic pain. The pathology may be in the cochlea, in the auditory nerve, or, most commonly, in the brain. Like chronic neuropathic pain tinnitus is not life threatening but influences many normal functions such as sleep and the ability to concentrate on work. Some forms of chronic tinnitus have two components, a (phantom) sound and a component that may best be described as suffering or distress. The pathology of these two components may be different and the treatment that is most effective may be different for these two components. The most common form of treatment of tinnitus is pharmacological agents and behavioral treatment combined with sound therapy. Less common treatments are hypnosis and acupuncture. Various forms of neuromodulation are becoming in use in an attempt to reverse maladaptive plastic changes in the brain.

## 1. Introduction

Sensorineural tinnitus is defined as hearing a sound that has no physical correlate; hence the sound of tinnitus is often described as being a phantom (ghost) sound.

Severe sensorineural tinnitus is a phantom sensation of sound that is often accompanied by an effect that is best described as suffering. In many ways, this form of tinnitus is similar to chronic neuropathic pain [1, 2]. Tinnitus has no visible signs of illness and is not life threatening, though it may affect quality of life and can cause suffering. A French surgeon, René Leriche (1879–1955), remarked, “The only tolerable pain is someone else's pain.” This would also apply to tinnitus.

Many forms of tinnitus have two main attributes. One is the sound the person hears and the other is the general effect the disease has on the person, best described as “distress” or “suffering.” The amplitude (or volume) of the perceived sound and the degree of suffering are not always directly related. The pathology of these emotional components which often accompanies tinnitus is not widely understood and this contributes to the lack of seriousness with which tinnitus is often considered by physicians and other health care personnel.

The pathology of tinnitus is poorly known, but the fact that the disease can occur in people with a severed auditory nerve shows that tinnitus can be caused by abnormal neural activity that is generated in the brain without the involvement of the ear. The phantom sensations in tinnitus and the effects on a person (suffering, distress, etc.) are similar to those of chronic neuropathic pain [1, 2]. Many kinds of tinnitus are similar to the phantom limb syndrome. In these disorders the symptoms are referred to a different part of the body than the anatomical location of the pathology.

Both chronic neuropathic pain and tinnitus include strong emotional components accompanied by affective “mood” disorders such as depression [3], but it is not known if successful treatment of the depression will have an effect on the tinnitus.

There is a lack of physically visible signs in disorders such as tinnitus and chronic neuropathic pain and there are no objective tests that can assess the strength of the symptoms or determine whether a person indeed has such symptoms. The fact that chronic neuropathic pain or tinnitus is not life threatening contributes to the lack of sympathy from relatives and friends and it lessens concern from physicians and other health professionals. Only the patient's own description is available in this regard. The patients' description of his/her tinnitus provides little information about the anatomical location of the pathology.

## 2. Characteristics of Tinnitus

It has been reported that a little over half of the people who have tinnitus perceive sound as coming from the ear; others perceive the sound as emanating from inside the head. A few people perceive the sound as coming from outside the head. Approximately 60% experience tinnitus bilaterally, while the remainder has unilateral tinnitus [4].

Almost all people with tinnitus have some form of hearing loss [5, 6], but some people have hearing loss and no noticeable tinnitus. The incidence of hearing loss and of troubled hearing loss increases monotonically with age. The incidence of bothersome tinnitus [7] lasting over 12 months also increases with age but plateaus between ages 65 and 75 after which it decreases slightly [8].

If more people who have tinnitus die earlier than people who do not have tinnitus, this would explain the observed decrease in the incidence of tinnitus at advanced age. That would occur if tinnitus has similar risk factors as diseases with high age-related mortality or if comorbidities of tinnitus have high age-related deaths.

## 3. Epidemiology

The prevalence of tinnitus reported by different epidemiological studies [11, 12] shows great variation. A study has reported that approximately 80% of adults have experienced tinnitus of some form, but the prevalence of severe chronic tinnitus is much less; reports show values from 4.1% to 15.1% [13]. Some of the variations are caused by differences in the criteria for having tinnitus. Studies agree that prevalence of tinnitus increases with age and that tinnitus is slightly more prevalent in men than in women.

The distribution of tinnitus among children has only been the aim of a few studies, but recent research found prevalence among children of 12–19 years to be similar or slightly higher than that of young adults (prevalence of tinnitus was 17.7% in this young population, although only 0.3% of the participants reported severe discomfort caused by tinnitus) [7].

## 4. The Effect of Tinnitus on a Person

For some people, tinnitus can be debilitating or unbearable, but for others, it is merely an annoyance. Some people with tinnitus also experience a general effect that is difficult to describe but perhaps is best termed as suffering. Tinnitus that causes suffering has also been described as “bothersome” or “troublesome” tinnitus [14, 15]. The suffering component of tinnitus is not directly related to the loudness of the sound of tinnitus. Some people may experience the fact that the loudness of their tinnitus is high without the tinnitus has much effect on their daily life, while others may have a tremendous reduction in quality of life from what they rate as weak tinnitus sound.

The loudness of the tinnitus sound is often assessed by using an analog scale (e.g., from 1 to 10), but more extensive questionnaires are used to assess the suffering component. The most commonly used questionnaires for assessing the effect of a patient's tinnitus are the Tinnitus Handicap Inventory, Tinnitus Handicap Questionnaire, and Tinnitus Reaction Questionnaire [16, 17].

These questionnaires are supposed to provide a measure of the degree of the handicap based on the loudness of the sound and the degree of annoyance and distress of the tinnitus sound and assessment of its effect on daily activities such as sleep, social interactions, and concentration as well as its effect on emotion and depression. These questionnaires, however, are self-report and the wording of the questions may influence the responses. Dwelling on that matter in the course of formulating an answer to a question may alter a person's perception of his/her tinnitus. The results must therefore always be evaluated in the context of the clinical impression and the patient's other tinnitus-related symptoms. This caveat is especially important for judging the effect of treatment. Use of only an analog scale for the loudness of the tinnitus is an incomplete measure of the effect of the treatment. The severity of the distress or suffering must be included in an evaluation of the outcome of treatment and selecting appropriate questionnaires is essential.

## 5. Pathology

The pathology of tinnitus is complex and poorly understood. There is evidence that an interplay exists between peripheral (ear) pathologies and central auditory mechanisms (auditory nervous systems and other parts of the brain) [18]. One of the obstacles in understanding the pathology of tinnitus is related to the diversity of diseases that exhibit similar symptoms, namely, phantom sounds of varying intensity and characteristics. Tinnitus research has in general been done under the assumption that tinnitus is a single disease with a few exceptions. Attempts have been made to differentiate between various forms of tinnitus, to a great extent by characterizing the symptoms such as which ear the tinnitus was referred to and the character of the sound which patients experienced. However, it has not been possible to relate these attributes to the structures in the brain that are involved in the tinnitus.

Functional imaging methods of various kinds have had limited success in distinguishing between the characteristics of the tinnitus and the anatomical locations of abnormalities. Studies of functional connectivity may have more specific power in distinguishing between different pathologies that can cause tinnitus [19].

In general, tinnitus research has paid great attention to the role of hyperactivity (increased firing rate) and hyperexcitability in structures of the auditory system. Animal experiments [20–22] have demonstrated that increased spontaneous neural firing rate and hyperexcitability in structures of the auditory nervous system can be caused by exposure to loud noise. Deprivation of sound input may occur in conductive or cochlear hearing loss. The mechanisms causing tinnitus are similar to those causing deafferentation pain. The resulting hyperactivity is thought to be the result of an increased central gain following peripheral denervation, which alters the balance between excitation and inhibition in a nervous system [23]. Hearing loss is also associated with hypersensitivity of structures of the auditory system [24, 25].

The fact that tinnitus can occur without input to the nervous system from the ear is evident from the findings that tinnitus occurs in people who have had their auditory nerve severed. These findings are similar to contemporary hypotheses about the pathology of chronic neuropathic pain [1].

Many forms of chronic tinnitus may be initiated by abnormalities in the ear or by exposure to loud sounds but, as time progresses, tinnitus tends to become independent of input from the ear. It is now generally accepted that this situation develops through functional changes brought about by activation of neuroplasticity. Neuroplasticity has generally been regarded as beneficial in that it is the basis for learning new skills and recovery from nervous system trauma [10], but plastic changes can be harmful and production of disease symptoms is less well known. Misdirected plasticity (maladaptive plasticity) is involved in many diseases of the central nervous system such as chronic neuropathic pain, spasticity, and some forms of muscle spasm, in addition to tinnitus. We have called such diseases “plasticity diseases” and these effects have been referred to as “the dark sides of neuroplasticity” [10].

Deprivation of auditory input to the brain is an important promoter of tinnitus through its activation of neuroplasticity. Hearing loss from middle ear problems results in reduced input to the brain from the ear and that may cause tinnitus by activating neuroplasticity. Deprivation of sensory input is the strongest promoter of neural plasticity [10]. Cochlear disorders can likewise cause such kind of deprivation of input to the brain from the ear.

There is now considerable evidence that activation of neuroplasticity is responsible for, or at least contributes to, many forms of tinnitus [26]. The central nervous system is, for the most part, plastic, and neural functions can adapt in response to external or internal factors.

The phantom limb syndrome where pain and other sensations are referred to an amputated limb is a clear example of a condition where it is obvious that the symptoms are generated by functional pathologies in the central nervous system; these symptoms could not have been elicited from the amputated limb to which the symptoms are referred.

The functional changes in the brain that are associated with tinnitus may involve many parts of the brain outside of the traditional auditory structures, such as limbic structure [27], especially the amygdala [28, 29]. Changes in the organization of the tonotopic map of the cerebral cortex may also be involved [30]. However, other investigators have presented conflicting evidence regarding the role of reorganization of tonotopic cortical maps in tinnitus [31].

Change in temporal integration that occurs in some forms of tinnitus [25] is a sign of abnormalities in central nervous system structures that are similar to that has been shown to occur in chronic neuropathic pain [1].

An old hypothesis assumes that synchrony (time locking) of neural firing in the auditory nerve is more important in signaling the presence of a sound than an increase in the discharge rate of nerve cells. Consequently, abnormal phase-locking in the auditory structures could cause tinnitus. This hypothesis was later supported by studies by Eggermont and other investigators [32]. Changes in coherence in large neural populations have more recently been suggested to be important in creating such diseases as severe tinnitus [33].

**Figure 1:**
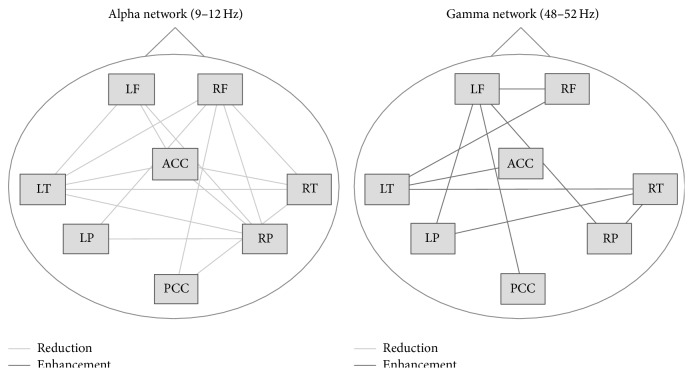
Schematic display of the alpha and the gamma network. Connections with a significant group difference were plotted as edges in the networks. The nodes were named as LF (left frontal), RF (right frontal), LT (left temporal), RT (right temporal), LP (left parietal), RP (right parietal), ACC (anterior cingulate cortex), and PCC (posterior cingulate cortex) (modified from Schlee et al., 2009 [9]).

Recent neuroanatomical studies have found that the evidence behind structural abnormalities associated with tinnitus is poor [34]. On the other hand, other studies have shown evidence that changes in functional connections in the brain play important roles in creating the symptoms of tinnitus [9, 35]. Functional connections differ from anatomical connections; functional connections are graded and depend on the efficacy of synapses. There is now considerable evidence of abnormal functional connections between brain regions in people with chronic tinnitus. Studies have demonstrated that the altered functional connectivity effects parts of the brain not normally associated with hearing [9] (Figure 1), such as the insula.

**Figure 2:**
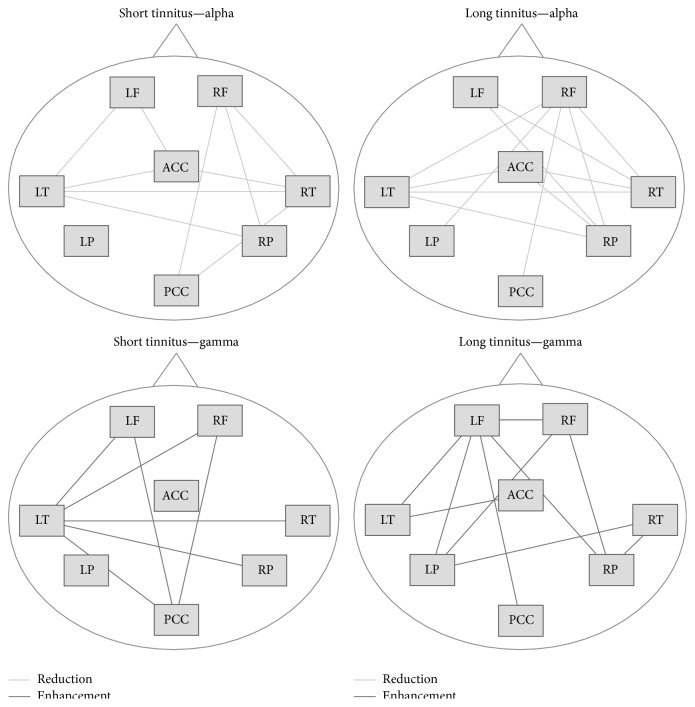
Schematic displays of alpha and gamma networks for persons with tinnitus of short and long duration. LF: left frontal, RF: right frontal, LT: left temporal, RT: right temporal, LP: left parietal, RP: right parietal, ACC: anterior cingulate cortex, and PCC: posterior cingulate cortex (modified from Schlee et al., 2009 [9]).

Not only does the functional connectivity in the brain of people who have tinnitus differ from that in people who do not have tinnitus but also the functional connectivity differs between people who have recently acquired tinnitus and people who have experienced tinnitus for a very long time [9] (Figure 2).

The results of studies of connectivity depend to some extent on which parts of the EEG spectrum are used. Using low frequency (alpha) components as the basis shows different connections compared to using high frequency (gamma waves) as seen in Figure 2.

## 6. Comorbidities

Tinnitus is one of the three symptoms that define Ménière's disease. People with vestibular neuroma almost always experience tinnitus. Some body disorders such as temporomandibular joint (TMJ) disorders [36, 37] and various kinds of neck problems are often accompanied by tinnitus [38].

There is evidence that abnormal activation of the dorsal-medial thalamus plays an important role in tinnitus, especially regarding the suffering component. This may explain some of the comorbidities of tinnitus. The subcortical connections from cells in the dorsal-medial thalamus bypass the primary cortices and connect directly to limbic structures such as the amygdala and the hippocampus [39, 40]. This may explain why the increased risk of depression has been shown to occur in tinnitus [3].

The dorsomedial thalamus may be involved in tinnitus through activation of the nonclassical (extralemniscal) pathways.

There is evidence that stress is involved in creation of tinnitus indicating a role of the sympathetic nervous system. The role of the immune system in tinnitus has received little attention, but studies of its role in chronic neuropathic pain indicate that it may also have a role in tinnitus [41–43].

The increased incidence of depression and other affective and psychiatric diseases [44] as comorbidity to tinnitus can be taken as sign of involvement of the limbic system. It is therefore important to perform a psychological or even a psychiatric assessment on patients with severe tinnitus [45].

Tinnitus is often accompanied by hyperacusis [46]. Hyperacusis is a lowered tolerance for sounds [46], but the term is often incorrectly taken to mean increased sensitivity such as recruitment of loudness. Hyperacusis may cause more distress than the perception of the tinnitus [47, 48]. Hyperacusis affects all kinds of sounds, but little is known about its pathology. Little is known about the pathology of hyperacusis. The pathology may not be in the auditory systems as such but rather in neural circuits that set the tolerance level of sensory signals.

Phonophobia is a rare symptom that may occur together with tinnitus. It involves fear of some kinds of sounds. Misophonia is a strong adverse reaction to very specific sounds, often sounds that are associated with eating [49, 50].

The term misophonia was introduced by M. Jastreboff and P. Jastreboff, 2001 [51], who suggested that misophonia may be a special case of phonophobia in which fear is a dominant emotion [51]. Since the symptoms misophonia are caused by very specific sounds, it seems likely that pathology in the central part of the auditory nervous system is involved in creating the symptoms of this rare disorder. The strong involuntary (autonomic) components of the symptoms suggest involvement of the limbic system or perhaps the insular lobe structures. It is not certain whether misophonia is a part of the tinnitus family or a completely separate disease.

Exploding head is a little known symptom that is characterized by a sensation of sudden, strong sound, often occurring during sleep and waking up a person [49, 52]. It may be regarded to be a form of tinnitus with similarities to audiogenic seizures [53]. The pathology of the exploding head syndrome is unknown.

## 7. Treatment of Tinnitus

Tinnitus is far more complex than was earlier assumed and many of the treatments now in use are experimental. Some are controversial and some have side effects [54]. There is a general consensus that a multidisciplinary approach to treatment is crucial for success. There are many different kinds of tinnitus and certain treatments are only effective for treating certain forms. A single treatment cannot be expected to treat all forms of tinnitus with success, which confounds the measure of that treatment's effectiveness. Regarding tinnitus to be a single disorder, as is often done, is perhaps the greatest obstacle in developing effective treatments for tinnitus. To accomplish that better diagnostic methods and probably different kinds of diagnostic methods have to be developed that can distinguish between the many different kinds of tinnitus. As the diagnostic methods to distinguish kinds of tinnitus are still limited the best way to find a treatment is often to try different treatments and see which one works.

In an attempt to distinguish between tinnitus of different pathologies, much attention has been given to the character of the tinnitus sounds through attempts to match a person's tinnitus sound to an actual physical sound but that has produced mixed results regarding treatment. The exact character of the tinnitus sound seems to have limited importance in the treatment of the condition, with the possible exceptions of discerning between tonal and atonal tinnitus and determining whether the tinnitus is of low or high frequency. It is possible that the tinnitus sounds do not generally have the character of any physical sound.

### 7.1. Treatment of Underlying Disorders 

Tinnitus is associated with many different treatable disorders. Some forms of tinnitus have underlying somatic disorders and successful treatment of these disorders is often effective in relieving the tinnitus.

When treating people with some forms of tinnitus, it may be beneficial to consider that many people with tinnitus have stress and psychological and psychiatric comorbidities [55] that interact with the pure symptoms of tinnitus [56]. Treatment of these conditions can often provide relief from tinnitus or make tinnitus less bothersome.

Tinnitus often occurs in people who take ototoxic medications such as some antibiotics, diuretics (Furosemide), and cancer drugs (e.g.,* cis*-platinum). Administration of furosemide can also have the opposite effect, namely, reducing tinnitus, probably because it reduces hyperactivity in the central nervous system [57].

Conductive hearing loss of any kind may be associated with tinnitus and tinnitus is often eliminated or reduced when these problems are resolved. Otosclerosis is often associated with tinnitus, which is relieved when the hearing loss is resolved by a successful operation. Hearing aids can increase the input to the nervous system and oftentimes reduce tinnitus [58].

Cochlear implants can relieve tinnitus in people who have severe hearing loss [59, 60].

People with vestibular Schwannoma almost always have tinnitus. Surgical treatment of vestibular Schwannoma rarely reduces or eliminates tinnitus but often exacerbates it. It is nonetheless important to rule out vestibular Schwannoma in all people who have tinnitus that is referred to one ear only and who have asymmetric hearing thresholds. Although vestibular Schwannoma often is the first manifestation in people with NF2, the disorder often has other symptoms that affect other cranial or spinal nerves.

Tinnitus often occurs together with temporomandibular joint (TMJ) problems or neck problems [37]. Correction of such pathologies can often reduce a person's tinnitus.

There is considerable evidence that stress can promote or exacerbate tinnitus [13, 56, 61] and there is also evidence that tinnitus can cause stress-like symptoms. Other studies [56] acknowledge that while stress has an influence on tinnitus suffering, stress alone cannot explain psychological comorbidity of tinnitus. This is similar to certain skin disorders such as psoriasis and many disorders of the digestive system in which stress plays a role in causing the symptoms but is not the single cause.

Diseases such as hypertension [62] affect the prevalence of tinnitus and it may be hypothesized that hypertension also increases the risk of tinnitus and that tinnitus promotes the development of hypertension.

### 7.2. Treatment of Subjective Idiopathic Tinnitus

Current treatments for idiopathic tinnitus include medication [4, 63, 64], neuromodulation [65], acupuncture, behavioral therapy [66], hypnosis [67], and masking [68].

Of the above methods, behavioral therapy seems to be the most successful [66], though the other methods, particularly neuromodulation, are now being developed at a fast pace and their clinical importance can be expected to grow rapidly [69].

The success of treatment of severe tinnitus depends on many factors such as the severity of the tinnitus, its character, and impact on quality of life as well as the patient's age, hearing status, and whether other diseases are present. Patients' compliance with treatment regimens is also essential for the success of any treatment. The outcome also depends on a patient's emotional state and how he or she reacts to the tinnitus.

Behavioral treatments do not depend on knowing the anatomical location of the pathology, but other treatments such as transcranial magnetic stimulation (TMS) or transcranial electrical (DC) stimulation or direct electrical stimulation of specific structures in the brain do.

Many people with tinnitus are convinced, erroneously, that there is something wrong with their ears; the pathologies behind most forms of tinnitus are in the brain. It is important to explain to such patients that altered function in some parts of the brain may imitate the distinctive neural signal of a bona fida sound and that such internally generated neural activity may therefore be interpreted as coming from the ear.* It does not help to treat the ear when the problem is in the brain!* The person who treats a patient with tinnitus may therefore have to describe in detail why tinnitus can be caused by changes in the function of a part of the brain.

Sometimes the most beneficial treatment is to focus on mitigating the suffering or distress from tinnitus that can affect a person's daily life rather than trying to decrease the loudness of the tinnitus. The measure of the success of treatment should therefore focus on the entire effect of the tinnitus on a person rather than focusing on the decrease in the perceived tinnitus sound.

It is always important to give the patients realistic hope for success in the treatment. Unfulfilled goals will result in disappointment and search for other health professionals who may promise full relief. Setting a reasonable treatment goal is also important. The absolute goal would be that the distress from the tinnitus and its resulting sound should be eliminated completely. This, however, is not often attainable practically. A more realistic goal would be the reduction of the tinnitus to a level where it is less burdensome (disease management).

Tinnitus has a different effect on different people and different people use widely different ways to describe their tinnitus and their concern. Some people are initially concerned that their tinnitus is a sign of a serious disease such as a brain tumor. Further tests such MRI scans can effectively rule out that possibility and relieve a person from fear of other serious diseases. After a person has been assured that his/her tinnitus is not a sign of a brain tumor, often no treatment is required.

### 7.3. Treatments with Medications

Many different substances have been tried, but few have been both effective and practical to administer and most of the suggested treatments have had more or less severe side effects [54]. Side effects are always a problem when pharmacological substances are used for treatment and treatment of tinnitus is no exception. Only a few drugs have shown enough beneficial effect that they are in clinical use for treatment of tinnitus. In that respect there is considerable contrast between tinnitus and pain, for which many effective pharmaceutical treatments exist [1]. Thus, there is an urgent need for development of more effective medications for treatment of tinnitus [69, 70] and it may be of value to learn from treatment of pain, especially chronic neuropathic pain that is otherwise considerably similar to tinnitus.

One of the first medications that was shown to suppress tinnitus is the local anesthetic, lidocaine (Xylocaine®), that when injected was shown to suppress tinnitus [71]. As it was only effective when administered intravenously; it never entered general use for treatment of tinnitus. Lidocaine has a somewhat complex action in that it under some circumstances can increase the loudness of the tinnitus. Initial expectations of success in treatment of tinnitus from administration of lidocaine have led to many attempts to develop effective medications [4, 70]. That would have similar beneficial effect without the need to be administered intravenously. Tocainide® is an example of a drug that is similar to lidocaine but can be taken orally. Unfortunately, Tocainide has an unsatisfactorily low therapeutic effect and severe side effects affecting the heart [63, 72, 73].

Alprazolam (triazolobenzodiazepine) is a short-acting substance that is used to treat anxiety, panic attacks, and depression. Alprazolam (Xanax®) is a member of the benzodiazepine family that has shown good effect on tinnitus [63, 68, 74]. Clonazepam (Klonopin®) is another member of the benzodiazepine family that is in general used as a muscle relaxant, anxiolytic, and anticonvulsant and which is in used for treating some forms of tinnitus [63]. A recent study found clonazepam to significantly decrease the loudness of the tinnitus sound in 75% of the participants in a study of an open-label, randomized, crossover study on 27 men and 11 women aged 16–80 (mean 58) [75].

Other studies have found that clonazepam on its own does not outperform the placebo until combined with Deanxit® (Flupentixol®) and Melitracen®, an antidepressant [76]. These two studies show how different investigators can come to very different results when drugs are tested in population studies. However, it is still not easily predicable which patients will benefit (for details about pharmacological substances that may have beneficial effects on tinnitus see Salvi et al. 2009 [4] and Langguth et al. 2009 [70]).

Some anticonvulsants may have a small beneficial effect on tinnitus [77], but this benefit has not been shown to be clinically significant. Anticonvulsants such as carbamazepine (Tegretol®) that are in general used for treatment of people with epileptic seizures and for treating trigeminal neuralgia and some other pain disorders [1] are sparsely used in treatment of tinnitus [63]. Another drug, sodium valproate (Depakene®) that is in general used for treatment of seizures, bipolar disorders, and mood disorders and depression has had some use in treatment of some forms of tinnitus. Gabapentin (Neurontin®) [78] and pregabalin (Lyrica®) are anticonvulsant drugs in limited use for the treatment of tinnitus. These two drugs are widely used in the treatment of seizures, neuropathic pain, and migraines, although their exact mechanisms of action are not understood.

Some drugs may affect specific aspects of tinnitus. For example, nortriptyline (Aventyl®) that is used to treat people with the chronic fatigue syndrome, migraine, and chronic neuropathic pain [1] seems to affect the mood components of tinnitus and in some individuals it also has a beneficial effect on the loudness of chronic tinnitus [79]. Nortriptyline is an antidepressant that inhibits the reuptake of norepinephrine and, to a lesser extent, serotonin. In a 12-week, double blind, randomized controlled trial nortriptyline significantly reduced depression scores and tinnitus loudness (10 dB reduction) [79].

Zinc and other supplements such as omega 3 have produced positive results in small studies, despite having nonstatistically significant effects in a randomized placebo controlled crossover trial [80].

Local application of drugs in the ear has had some success in treating tinnitus. Intratympanic dexamethasone is an example that has shown beneficial effect in some persons with tinnitus [81], but other drugs have also been used [82].

More recently several medications are either under development or advanced to the state of clinical trials. One example is an NMDA-R antagonist, gacyclidine, by the company Otonomy. Gacyclidine is a phencyclidine derivative that has neuroprotective properties. In a study in 6 patients with unilateral deafness and tinnitus it has been shown to provide temporary relief of tinnitus in 4 of 6 patients when administered locally to the cochlea [83]. Another drug, a steroid dexamethasone, is under development by the same company for treatment of Ménière's disease. The company, Auris Medical, has another NMDA agonist, Esketamine hydrochloride, in phase 3 clinical development for treatment of tinnitus. The target for this medication is acute inner ear tinnitus such as that which occurs from acoustic trauma as a result of exposure to loud noise.

Recently the company Sound Pharmaceuticals are testing drugs such as an oral formulation of ebselen [84] for protection of the ear and thus reducing the risk of tinnitus from various kinds of insults to the cochlea. For other drugs that may have beneficial effect for people with certain forms of tinnitus, see Salvi et al., 2009 [4].

### 7.4. Surgical Treatment of Tinnitus

Particular forms of tinnitus have been treated successfully by microvascular decompression operations [85–87]. Microvascular decompression operations of the auditory nerve are the only direct surgical treatment of idiopathic tinnitus that is in general use.

### 7.5. Neuromodulation

Several forms of neuromodulation are entering use for the treatment of tinnitus [65]. Sound stimulation accomplished by the use of hearing aids or electrical stimulation of the ear through the use of cochlear implants can compensate for the deprivation of input that caused the tinnitus [59, 60]. Additional methods include electrical stimulation of the central nervous system through transcranial magnetic stimulation (TMS) [88, 89], through transcranial electrical (DC) stimulation [91], or through implanted electrodes on the cerebral cortex [92] and other structures of the auditory nervous system.

Neuromodulation in the form of electrical stimulation of the somatosensory system has been used for many years for treatment of some forms of pain [1]. Actually, electric stimulation was used for the treatment of headaches in the 1st century and electrical stimulation was used in the 19th century though it was taken over by quackery [93].

It was probably stimulation of parts of the somatosensory system that was the first systematic use of neuromodulation for treatment of tinnitus. It was shown that electrical stimulation of specific peripheral somatosensory nerves can modulate tinnitus in people with some forms of tinnitus. Success with direct stimulation of the surface of the cochlea [88, 94, 95] has inspired a less invasive treatment in which a similar effect is achieved through electrical stimulation of the skin behind the ears. It was believed that the applied electrical current could reach the cochlea and thereby reduce a person's tinnitus. However, despite its beneficial effect in some people with tinnitus, the method was discarded because it seemed improbable that enough electrical current would reach the ear (cochlea) by such stimulation.

Later it was found that that fibers of the C_2_ spinal dorsal root that innervate the skin behind the ears also target the cells in the dorsal column nuclei that send axons to cells in the dorsal cochlear nucleus. This is probably the manner in which electrical stimulation of the skin behind the ears suppresses tinnitus [96]. The early findings that electrical stimulation of the surface of the cochlea could reduce tinnitus [88, 94] could equally well be explained by stimulation of the C_2_ afferent fibers, which also innervate the mucosa of the middle ear cavity.

Acupuncture [97] that stimulates the somatosensory system can be regarded as a form of neuromodulation. Low-level laser therapy has also been tried for treatment of tinnitus and found to have a beneficial effect on some forms of tinnitus [54, 98].

Electrical stimulation of the vagus nerve (vagus nerve stimulation, VNS) is now being studied extensively for treatment of tinnitus and for enhancing reversal of neuroplastic changes that are assumed to have caused a person's tinnitus [99–101]. For that VNS is done together with presentation of sounds that are assumed to act against the tinnitus [99–102].

**Figure 3:**
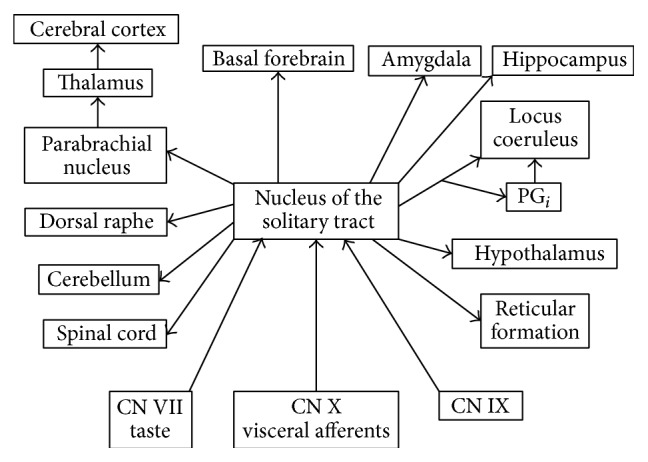
Targets of axons from cells in the nucleus tractus solitarius. Composite of different investigators description of the pathways from the NTS. PGi: Paragigantic nucleus (from Møller, 2014 [10]).

The vagus nerve is a part of the autonomic nervous system and is involved in the regulation of metabolic homeostasis, playing an important role in the neuroendocrine-immune axis (anti-inflammatory pathway) [103]. The descending fibers of the vagus nerve supply parasympathetic control to the heart and organs in the abdomen. The afferent vagus nerve fibers target cells in the nucleus of the solitary tract (NST), which send axons to many different parts of the brain (Figure 3). Of importance to the treatment of tinnitus is the fact that these axons can activate cells in the nucleus of Meynert and thereby facilitate plastic changes. VNS therefore offers a unique possibility of enhancing neuroplasticity.

VNS has been used in treatment of some forms of pain [104] and it has been used for many years in the treatment of epilepsy [105] (FDA approved 1997). VNS is used in an attempt to reverse maladaptive plasticity that is assumed to be involved in diseases such as tinnitus and chronic neuropathic pain [10].

The vagus nerve also exerts control over the immune system through the vagal immune reflex [54, 106].

### 7.6. Hypnosis

Hypnosis can be regarded as a form of neuromodulation found to be beneficial by some individuals effected by tinnitus. Hypnosis can actually produce functional changes in many parts of the brain. For example, hypnosis can produce general analgesia and it has been found effective in treatment of some forms of pain (see [1]).

### 7.7. Behavioral Treatment

Cognitive behavioral therapy (CBT), which aims at treating a person's reaction to tinnitus, has been shown to be an effective tool in the treatment of many forms of the disease [54, 107, 108].

Tinnitus retraining therapy (TRT), which can be regarded as a form of behavioral treatment is a well-established treatment of tinnitus that has been reported to have long-term improvement in tinnitus in 80% of patients who were treated [109]. P. J. Jastreboff and M. M. Jastreboff [110] developed TRT based on a neurophysiological model of tinnitus originally published by Jastreboff [48]. It involves directive counseling and sound stimulation using specific sounds [111]. TRT has produced excellent long-term results though tinnitus masking is more effective in the short term [112]. Another study [113] found that both TRT and general counseling without the additional sound therapy were both effective in reducing the annoyance component and personal impact of tinnitus. TRT offered the largest effect on tinnitus handicap (effect size of 1.13). The control group, however, also had a clinically significant effect (effect size of 0.78). This means that the placebo effect is large.

### 7.8. A Person's Reaction to Tinnitus Is Important for Treatment

**Figure 4:**
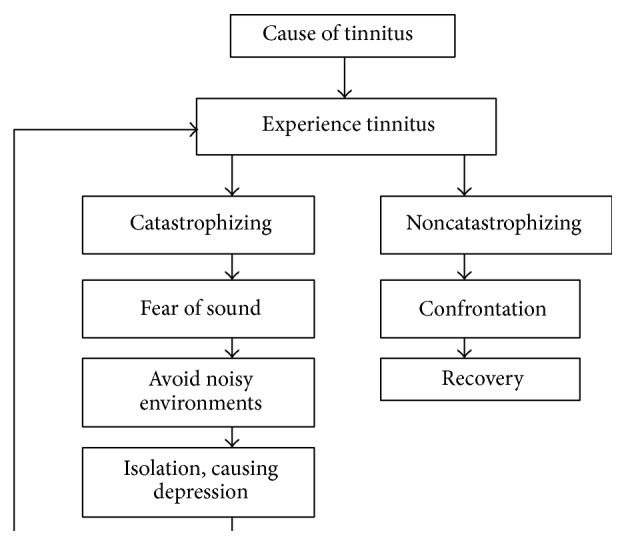
Hypothetical description of the difference between catastrophizing and noncatastrophizing in tinnitus (from Møller, 2014 [10]).

A person's reaction to his/her tinnitus has a tremendous impact on the outcome of any treatment. A person can have essentially two different reactions to tinnitus [114, 115]. One option is to catastrophize—that is to overreact, exaggerate, or imagine the worst situation [116]. The second option, which is typically more beneficial, is to confront the situation (Figure 4). Ellis [117] introduced the term catastrophizing and Beck et al. [118] later adapted the term to describe a “maladaptive cognitive style” used by people with anxiety and depressive symptoms. Different writers have described catastrophizing in different ways.

A noncatastrophic approach involves confronting the situation, perceiving the issue to be no greater than it actually is.

Catastrophizing statements may induce or reinforce tinnitus. If a person with tinnitus repeatedly hears or reads a statement such as “I cannot sleep because of my tinnitus,” the person may become convinced that his/her insomnia is caused by the tinnitus. Though the insomnia may be caused by another factor, if the tinnitus patient is convinced that the insomnia is linked to the tinnitus, he or she may retain the insomnia even after the actual cause is removed.

Coping is a learned beneficial skill that can reduce suffering [119]. The opposite, maladaptive coping strategies (e.g., statements about suicide) may increase the distress experienced from tinnitus. Studies have identified the specific brain regions that are involved in such maladaptive coping [120].

### 7.9. Development of New Treatment of Tinnitus

An examination of the studies of new medications, supplements, or other forms of treatment reveals that some treatments have a small effect, not significantly larger than that of the placebo. Evaluations of treatment results for tinnitus are hampered by the diversity of the disorder and inability to distinguish between patients with different tinnitus disorders. This means that a cohort of patients with tinnitus may show moderate results to a particular treatment while specific groups of patients within the cohort may show much better results. If, hypothetically, a cohort that is tested comprises three different tinnitus disorders with equal (33.3%) representation and in which the treatment that is tested is only effective in the treatment of one of these disorders, the success will never exceed 33% despite 100% success for one of the tinnitus disorders. If the treatment is only effective in 50% of these participants the total measured success rate will be 16%, still probably acceptable, but the placebo effect could be similar and such a treatment would be regarded as unacceptable.

The results of not being able to test treatments on groups of people with the same form of tinnitus may have excluded treatments that would have been of value if there would have been a possibility to distinguish between different tinnitus disorders.

Another problem in evaluation of treatment results is related to the fact that tinnitus has two different parts, the loudness of the phantom sound and the general effect on a person that is best described as suffering. Treatments may have different effects on these two components; a treatment may significantly lower the suffering component with little effect on the loudness of the sound. Treatments that affect suffering without causing much change in loudness may have been regarded as ineffective as treatment of tinnitus.

The placebo effect is large in tinnitus and that may have caused treatments that have no or little beneficial effect to be chosen.

## 8. Conclusions

Subjective tinnitus often has two different kinds of symptoms, one is the commonly recognized phantom sound and the other is what may best be described as suffering. The phantom sound and the suffering from tinnitus may engage different parts of the brain and tinnitus in general may engage different parts of the brain than those activated when physical sounds reach the ears. There is evidence that an interplay between peripheral (ear) pathologies and central auditory mechanisms (auditory nervous systems and other parts of the brain produce the symptoms of many forms of tinnitus). Activation of maladaptive neural plasticity is regarded to play a role in creating the pathologies that are associated with severe tinnitus. The phantom sound of tinnitus, the suffering, and other accompanying symptoms may be caused by the creation of abnormal connections in the brain involving an increased influence of the dorsomedial thalamus, which has direct connections to subcortical structures such as the amygdala, the anterior cingulate, and the insular lobe.

Often the suffering has been associated with the effect of the phantom sound, but there is now evidence that the abnormal neural functions that causes the suffering component of tinnitus is anatomically different from that which causes the phantom sound.

Perhaps the most effective treatments of tinnitus are different forms of behavioral treatments. Treatments using pharmacological agents have had moderate success and there are now efforts to use other methods. Various forms of neural modulation seem to be promising alternatives to pharmacological treatments. Since many forms of tinnitus are assumed to be caused by activation of maladaptive plasticity forms of “unlearning” tinnitus are now being studied in a search for an effective treatment for some forms of tinnitus. More specific forms of “unlearning” tinnitus are now being studied in a search for effective treatments for some forms of tinnitus. In any form of treatment, a person's reaction to his/her tinnitus is important for the success of treatments. A person with tinnitus may either confront the condition or catastrophize it.

In any form of treatment, a person's reaction to his/her tinnitus is important for the success of treatments. A person with tinnitus may either confront the condition or catastrophize it.

The poor success of treatment is, to a large extent, a result of not distinguishing between multiple kinds of tinnitus. The state of tinnitus treatment seems be comparable to that of cancer treatment in 1971 when the National Cancer Act launched the “War on Cancer.” The recent increase in success of treatment of cancer is to a large extent a result of realizing that cancer is not a single disease but a group of (very) different diseases. Should tinnitus be regarded in a similar way as a group of diverse diseases, we are likely to see a similar increase in the success of developing powerful and effective treatments for individuals affected by the tinnitus diseases.
